# Profile and Outcome of Childhood Hydrocarbon Poisoning: An Observational Study

**DOI:** 10.7759/cureus.20144

**Published:** 2021-12-03

**Authors:** Karthika IK, Debashree Priyadarshini, Swathi Nakka, Joseph John, Samarendra Mahapatro, Bhagirathi Dwibedi, Amit K Satapathy

**Affiliations:** 1 Pediatrics, All India Institutes of Medical Sciences, Bhubaneswar, Bhubaneswar, IND; 2 Pediatrics, All India Institute of Medical Sciences, Bhubaneswar, Bhubaneswar, IND

**Keywords:** pediatric, poisoning, kerosene, hydrocarbon, observational study

## Abstract

Background: Accidental poisoning, though preventable, has continued to be one of the most prevalent medical emergencies among children in developing countries.

Objective: To describe the clinico-demographic profile and outcome of children with hydrocarbon poisoning at a tertiary care teaching hospital from Eastern India.

Methods: A retrospective analysis of the hospital case records of all children <15 years of age admitted with hydrocarbon poisoning from January 2015 to October 2018 was performed.

Results: Out of 2658 total admissions, 21 children were admitted with hydrocarbon poisoning during the study period. The median age was 2.6 years (range, 1 to 14 years). Majorities (76%) were <5 years of age. The male/female ratio was 5:2. The majority belonged to rural areas (80.9%) and low socioeconomic status (71.4%). In all the cases, poisoning was accidental in nature. Kerosene (71.4%) and turpentine oil (24%) were the most common agents implicated. Majorities (95.3%) were symptomatic requiring hospitalization. Fever and vomiting were the most commonly observed symptoms (57%). Neutrophilic leucocytosis was observed in 62% of cases. Abnormal chest radiography was observed in 67% of cases. There was no mortality.

Conclusions: The majority of the children with hydrocarbon poisoning are under five years of age with the accidental mode of poisoning in the current study. Kerosene was the most common agent. The outcome was excellent without any mortality.

## Introduction

Childhood poisoning is regarded as one of the most important pediatric emergencies worldwide [1]. It is also considered the most preventable cause of mortality and morbidity in pediatric patients [2]. An annual estimate of 193,460 deaths as per the World Health Organization (WHO) report in 2012 are caused due to unintended poisoning all over the world [3]. Around 84% of this estimate is accounted for by low medium-income countries nations. Acute poisoning in children has a variable clinical presentation and outcome in different parts of the world. The prevalent socio-economic factors, occupational modalities, traditional beliefs, and the type of health care services in a given region are known to influence the pattern of poisoning as well.

Most of these poisoning cases result from exposure to commonly encountered, nontoxic domestic products in developed countries [4]. This is attributed to the decrease in exposure to toxic chemicals as a result of the implementation of strict child safety measures. The scenario is quite different in developing countries.

Kerosene has been implicated as the most prevalent cause of accidental poisoning in the pediatric age group, as per studies conducted on children from various countries including India [5-7]. The major cause seems to be the incessant use of kerosene oil as fuel for cooking in rural and urban slum areas. Boys are predominantly affected, particularly under five years of age because of their exploratory nature and maximum activity [5-8]. There is a scarcity of studies describing the profile and outcome of children with hydrocarbon poisoning from the Eastern part of India. So, the present study was undertaken to describe the epidemiology, clinical features, radiological picture, and treatment outcome in such cases.

## Materials and methods

The data were collected for a period of 2 years 10 months at a tertiary care teaching hospital in Eastern India from January 2015 to October 2018. The case records of all admitted children with acute hydrocarbon poisoning during this period were retrieved and analyzed retrospectively. The study population comprised of all the admitted children less than 15 years of age. The data extraction form was designed to collect demographic details including age, sex, nature, amount of poison ingested, and residency. Details of clinical presentation (symptoms, signs, time of onset of symptoms/signs, time of presentation to the emergency department) were recorded in the proforma. Laboratory investigations (complete blood counts, chest X-ray), treatment (supportive care, antibiotics, oxygen, invasive or non-invasive respiratory support), and outcomes (discharge, death) were collected from the records. 

All children presenting with acute hydrocarbon poisoning to our hospital were admitted and treated with a standard protocol. Initial management included ensuring adequate care of the airway, breathing, and circulation. Skin decontamination, maintenance of oxygenation, fluid and electrolyte balance, and ventilation were considered wherever needed. Monitoring was carried out as per standard guidelines. Chest X-ray and blood investigation including complete blood count, liver function test, and kidney function was done within the first 24 hours of admission. Repeat chest X-ray was done only if clinically indicated. Antibiotics were added only for children with respiratory distress and neutrophilic leukocytosis. Descriptive statistics, frequencies, and mean ± SD were used for data presentation.

## Results

During the study period, a total of 21 children (71% boys) were admitted with acute poisoning out of 2568 total admissions. The median age of presentation was two years six months (range, 1 - 14 years). The majority of the children (76%) were below five years of age followed by 6 to 10 years (19%), and 10 to 14 years of age (5%). The majority belonged to rural areas (80.9%) and were of low socioeconomic status (71.4%; Table 1).

**Table 1:** Demographic characteristics of the study population

Characteristics	Number (%)
Sex	
Boy	15 (71.5)
Girl	6 (28.5)
Age	
0–5 yrs	16 (76)
6–10 yrs	4 (19)
11–14 yrs	1 (5)
Socioeconomic status	
Upper	-
Middle	6 (18.5)
Lower	15 (71.5)
Season of presentation	
Summer	11 (52.3)
Rainy	2 (9.5)
Winter	4 (19.1)
Spring	4 (19.1)
Nature of poison	
Kerosene	15 (71.4)
Turpentine	5 (24)
Naphthalene	1 (4.6)

In all the cases, poisoning was accidental in nature. The mode of poisoning was ingestion only. The most common poisonous agents identified were kerosene and turpentine oil, accounting for 71.4% and 24% cases, respectively. Naphthalene poisoning occurred in 1 (4.6%) cases.

The average time of presentation to the emergency room was 24 hours (range, one hour to five days). It was longer for rural areas (24 hours) than urban areas (four hours).

Table 2 describes the clinical presentation of the admitted children. Only one patient (4.7%) was symptom-free after 12 hours of observation; remained 20 of the admitted patients (95.3%) were either symptomatic at the time of admission or developed so during the admission. Fever and vomiting were the most commonly observed symptoms (57%). Cough and breathing difficulty/respiratory distress was noted in 38% of cases. A serious symptom like a seizure was detected in one patient (4.6%).

**Table 2:** Clinical features at presentation

Symptoms	Number (%)
Fever	12 (57)
Vomiting	12 (57)
Cough	8 (38)
Breathing difficulty	8 (38)
Pain abdomen	7 (33)
Constipation	1 (4.6)
Seizure	1 (4.6)
Average time of presentation	24 hrs (range, 1 hr to 5 days)
Mean duration of hospital stay	5 days (range, 1 to 10 days)

Routine investigations (complete blood count, electrolytes) were done in 20 patients (95.2%). Evidence of neutrophilic leucocytosis (mean ± SD; 18,724 ± 5532/cmm) was observed in 13 patients (62%) along with an increase in C-reactive protein (CRP) level (>1 mg/dl). Chest X-ray was performed in 19 patients (90.5%). Features of pneumonitis were seen in 12 (57%) and consolidation in 1 (4.6%). One child developed pleural effusion as evident on a repeat chest X-ray done on hospital day 5. Chest X-ray was normal in 33% of cases (Table 3). The National Poison Information Center, AIIMS New Delhi was contacted for most of the patients and standard practice was followed. None of the patients required any specific antidote.

**Table 3:** Radiological features

Radiograph findings	Number (%)
Pneumonitis	12 (57)
Consolidation	1 (4.6)
Pleural effusion	1 (4.6)
Normal	7 (33)

Only one patient remained asymptomatic and was discharged 24 hours after observation. Remainder 20 (95.2%) patients required symptomatic treatment. Two children (9.2%) required ICU care in view of respiratory distress, and six (28.6%) required oxygen therapy. Eight children (38%) required antibiotics in view of respiratory distress with fever and neutrophilic leucocytosis along with elevated CRP. The mean duration of hospitalization was five days (range, 1 to 10 days). All patients were discharged, and there was no mortality. All children are doing well on follow-up.

## Discussion

Drug and chemical poisoning are considered to be the most commonly reported cause of unintended injury in pediatrics. It accounts for 0.33-7.6% of the total pediatric admissions at different health care centers in India [9]. This could be an underrating of the real burden of the poisoning cases as many are likely unreported.

Strong age predilection has been observed in cases of unintentional poisoning. Toddlers and children under five years of age are particularly vulnerable. The inquisitiveness to explore the surrounding environment and greater movement in this age group are mostly considered responsible for this. A strong oral orientation has also been observed in these children. The majority of children in our study belonged to those under five years of age which has also been reported in other studies [4]. Male preponderance seen in this study has also been in accordance with another study [10].

Most of the poisoning cases in children less than six years are usually accidental [11], which is also described in our study. Poisoning can be suicidal in adolescents and very rarely homicidal as a form of child abuse [11].

A major chunk of our patients belonged to rural communities. The median time of presentation after exposure to the toxin was 24 hours for children coming from villages as compared to those coming from urban areas, who presented within four hours. However, in another study, this time was comparatively shorter [12]. This is attributed to the longer distance travelled by the rural patients and also the primary care received by these patients at other peripheral hospitals, before their referral to our hospital.

Previous studies in India have attributed kerosene to be responsible for 25-50% of all childhood poisonings [12], which is well represented in our study (47.2%). Kerosene is mostly used by low-income families with improper storage in empty bottles on the floor, within the easy reach of children. The appearance of kerosene resembling other beverages might have prompted young children to drink it unintentionally.

This study indicates that a greater number of cases occurred in the summer season and a higher incidence of cases was noted in spring and rainy seasons as compared to winter, similar to a South African study [13]. During the summer season, the child can mistake kerosene for water or some cold drink to quench his thirst [13].

Supportive care was the mainstay of treatment. Antipyretics, intravenous fluids, electrolytes were administered to all patients and oxygen supplementation was given as and when required. Intravenous antibiotics were started on suspicion of secondary bacterial infection like persistence of high-grade fever, leucocytosis, high CRP, and deteriorating clinical condition. In our patients, eight received antibiotics in view of worsening respiratory distress and neutrophilic leucocytosis. Only one child had developed pleural effusion on day 5 which has been also reported as a delayed complication of kerosene poisoning. Pneumothorax, pneumomediastinum, and subcutaneous emphysema are the other complications that have been reported following kerosene poisoning [14]. Critically sick patients (9.5%) were transferred to the pediatric intensive care unit (PICU). However, no deaths were reported, similar to previous studies [4-7].

The retrospective nature and small sample size of the study are the major limitations of the study. This explains the non-availability of data on some of the crucial aspects.

## Conclusions

Hydrocarbon poisoning is one of the important causes of acute poisoning causing morbidity and mortality in children. The majority of the children with hydrocarbon poisoning are under five years of age with the accidental mode of poisoning. Kerosene was the most common chemical agent. Following standard protocol for the management of acute hydrocarbon poisoning prevents unnecessary investigations and the use of antibiotics. Antibiotics are only indicated in the presence of secondary bacterial infection. Early presentation to the hospital and strict monitoring during hospitalization results in excellent outcomes without any mortality. Increased parental supervision is the need of the hour to prevent such accidents.
